# Current News and Opinions

**Published:** 1888-11

**Authors:** 


### Current News.

### DOMESTIC CORRESPONDENCE.

*Editor Independent Practitioner:*

In your editorials in the August and September numbers in discussing the question of "Dentistry a Specialty in Medicine," you make use of the word profession several times; we would respectfully query what profession?

Every attentive reader of the dental and medical literature produced during the last five years will recognize the pertinent force of the above query.

From the time that Chapin A. Harris and his coadjators made overtures to the medical colleges, endeavoring to have dental professorships incorporated in them, and the scornful rejection of their propositions up to about 1883, dentistry was not confounded in thought with medicine by any one. It was understood to to have its own literature, colleges, societies, etc.; and the medical fraternity lost no opportunity to give prominence to the fact that dentistry had nothing to do with medicine. Dentistry as an independent profession, however, thrived and grew, and made for itself so prominent a position that its respectability and strength were felt far and wide. Medicine, ever jealous of her supposed prerogatives, has noted the healthy pulse, and during the last five years has made great change in her attitude and from her original policy. During this period a persistent attempt has been made by the medical profession to have it understood that dentistry is a specialty in medicine. This effort has filled our literature with much interesting reading bearing upon the subject, but has in no wise been able to make dentistry a specialty in medicine; but, rather, has continually emphasized the fact that the healing art is in the hands of *two great distinct and separate branches—medicine and dentistry.* Each distinct and independent. This letter is written with the view of calling attention to this emphasis.

Every article that has been written, every speech that has been uttered, advocating dentistry as a specialty in medicine, is filled with the expression, "the profession." Such emphasis shows how deeply implanted dentistry is as a profession.

Did space permit I would enumerate and quote from them for the edification of the general reader, but a few taken from your late editorials will suffice.

The italics are ours.

"No one at the present day questions the position of dentistry as a branch of the healing art, and as such a *specialty* in medicine." * * * "The individual members of the *profession* who have done most to secure recognition for the body corporate have been liberally or medically educated men." * * * "It was through the efforts and personal standing of these men that the *profession* was seated as a body in the American Medical Association." * * * "The American Medical Association did not elect to issue certificates of proficiency in obstetrics to those members of *the dental profession.*' * * * "This question is one of very considerable interest to us as a *profession.*"

In speaking of the action of the American Medical Association, you say: "To turn our backs upon them now would be unappreciation, to say the least." It would be hard to tell what is to be appreciated. Certainly not as you put it, "the overlooking of our deficiencies;" certainly not the attempt to garner the glorious work of so many dental years into the medical storehouse, and to obtain credit for the crop. Truly they seem desirious to "reap where they have not sown." Every one who has taken the pains to test the action of the American Medical Association and the Ninth International Medical Congress, knows the questionableness of the "we have been most magnaminously recognized as specialists in medicine." An adoption is no adoption which "does not establish us as such in the eyes of the law!"

The statement, "Step by step, one after another of the medical branches have been incorporated into the dental curriculum, until now it is considered essential to employ largely medically educated men to instruct in those branches," is inaccurate. The medical branches, so called, were all, and at once, placed in the dental curriculum when the first dental college was established, and have been retained ever since.

In conclusion, we must say that it seems a little peculiar and without reason to warn against the practice of implantation by those holding only the title of D. D. S. The operation is one purely dental in origin and in character, and one that is, and only can be, performed by dentists. This word of caution is finished with "thus are the necessities for broader education being constantly brought to the notice of *the profession.*" We would query, What profession?

Respectfully yours, L. ASHLEY FAUGHT, D. D. S.,

Oct. 11, 1888. 1435 Arch Street, Philadelphia.

### MEDICAL APHORISMS.

A correspondent, signing himself "Artz," sends to the *Canada Lancet* the following professional aphorisms of Amédèe Latour:

(1) Life is short, patients fastidious, and the brethren deceptive. (2) Practice is a field of which tact is the manure. (3) Patients are comparable to flannel—neither can be quitted without danger. (4) The physician who absents himself runs the same risk as the lover who leaves his mistress—he is pretty sure to find himself supplanted. (5) Would you rid yourself of a tiresome patient, present your bill. (6) The patient who pays for his attention is but exacting; he who does not is a despot. (7) The physician who depends on the gratitude of his patient for his fee is like the traveler who waited on the bank of a river until it finished flowing, so that he might cross to the other side. (8) Modesty, simplicity, truthfulness!—cleansing virtues, everywhere but at the bedside; there simplicity is construed as *hesitation*, modesty as *want of confidence*, truth as *impoliteness*. (9) To keep within the limits of a dignified assurance without falling into the ridiculous vauntings of the boaster constitutes the supreme talent of the physician. (10) Remember always to appear to be doing something—above all, when you are doing nothing. (11) With equal, and even inferior talent, the cleanly and genteelly dressed physician has a great advantage over the untidy one.

---

### ODONTOLOGICAL SOCIETY OF PENNSYLVANIA.

On the occasion of its tenth anniversary the Odontological Society of Pennsylvania will hold a two days' session, commencing at 2 o'clock on Wednesday, December 12th. The programme will consist of addresses and essays upon subjects pertaining to dentistry, which will be especially prepared for the occasion by leading members of the profession. A series of clinical demonstrations will also be given by a number of gentlemen having new features to present relating to special methods of dental practice.

An interesting exhibit will also be made by dealers in and manufacturers of dental goods, who will be afforded an ample opportunity to show the latest improvements in their respective lines.

The committee having the matter in charge have determined to make the occasion a notable one, and no pains will be spared to render the meeting interesting and attractive. A general invitation is hereby extended to the dental profession to meet with us and take part in the exercises and discussion of papers. The programme will be issued at an early date.

H. C. Register,
Chairman Anniversary Committee.

---

The price of Dr. Wardwell's very useful corrugated rubber foot pad for the tread on dental engines has been reduced from $1.50 to $1.00 each. We are sure that at this moderate price no dentist can find excuse to do without it. Those who have used them say that they would not be without them for any price.—Ed.

### A COLLAPSED DRUGGIST.

"I want some consecrated lye," he slowly announced, as he entered the store.

"You mean concentrated lye," suggested the druggist, as he suppressed a smile.

"Well, maybe I do. It does nutmeg any difference. It's what I camphor, anyhow. What does it sulphur?"

"Eighteen cents a can."

"Then you can give me a can."

"I never cinnamon who thought himself so witty as you do," said the druggist, in a gingerly manner, feeling called upon to do a little punning himself.

'Well, that's not bad, either," replied the customer, with a syruptitious glance. "I ammonia only a novice at the business, though I've soda good many puns that other punsters reaped the credit of. However, I don't care a copperas far as I am concerned, though they ought to be handled without cloves till they wouldn't know what was the madder with them. Perhaps I shouldn't myrrh-myrrh. We had a pleasant time, and I shall caraway—"

It was too much for the druggist. He collapsed —*Detroit Free Press.*

---

Resolutions Passed at the American Dental Association.—Resolutions offered by Dr. Marshall, Chicago.

*Whereas*, It is the sense and belief of the American and Southern Dental Associations, in joint meeting assembled, that the tariff on imported dental and surgical goods works a hardship upon the profession and the public; therefore, be it

*Resolved*, That we memorialize Congress to abolish all duties upon imported dental and surgical instruments, apparatus and supplies.

*Resolved*, That the secretaries of the Associations be instructed to forward these resolutions to the proper authorities in Congress, and that each member of the Association be requested to use his influence with the Congressman of his district to vote to place the above-mentioned goods upon the free list.

---

The Twenty-sixth Annual Meeting of the New England Dental Society will be held in Boston, Thursday and Friday, November 15 and 16, 1888. An interesting program has been arranged by the Executive Committee. Mark off the days *now* from your appointment book, and be sure and be with us.

Yours, etc., A. H. Gilson, *Secretary.*

---

The Twenty-fifth Annual Meeting of the Connecticut Valley Dental Society will be held at Springfield, Mass., December 6 and 7, 1888.

George A. Maxfield, D.D.S., *Secretary.*
Holyoke, Mass.

---

If there are any of our subscribers who are suffering from dyspepsia, and who feel the need of some form of tonic, we can most cordially recommend "Golden's Liquid Beef Tonic." It is easily digested and forms a very palatable and beneficial preparation.

### A NEW ANTISEPTIC SOAP.

Until quite recently a satisfactory soap containing as an antiseptic one of the salts of mercury, has been difficult to prepare on account of the alkaiine soap refusing to yield a good lather, oleate of mercury being formed—a compound which has little or no germicidal action. One of the most powerful of the mercury salts is, as is well known, the bichloride. Moreover, it is cheap and easily soluble, but it has the disadvantage of being extremely poisonous and easily reduced by albuminoid matter, with which it combines, thus being rendered inactive. In a paper recently read before the Society of Chemical Industry, by John Thomson, the solubility of the red biniodide of mercury (which is claimed to be even a more powerful antiseptic than the bichloride) in iodide of potassium has been made use of. A soap can thus be easily prepared containing a certain proportion of the biniodide in a soluble form. It is stated to be permanent, having no tendency to separate, and to be more germicidal in its properties than any other antiseptic soap yet known. Experiments were made to demonstrate this. In experiments carried out in the same manner with other antiseptic and ordinary soaps, it was shown that the growth of the organisms in many cases was not prevented. The importance of such a soap in medical and sanitary science is very obvious. The biniodide soap has been used in the treatment of eczema with well-marked success, especially where the irritation is due to the fermentation of accumulated secretions, the fermentation being set up by micro-organisms. It has also met with similar success when used in parasitic skin diseases, such as favus and ringworm. As a parasiticide, too, the importance of its application to patients during the period of desquamation in scarlet fever is evident. —*Lancet.*

---

### LIQUEFIED CHLORIDE OF METHYL AS A LOCAL ANÆSTHETIC.

At a recent meeting of the Societé de Biologie, M. Gallippe (*Bulletin Médical*, No. xi, 1888) stated that for the last two years he had been employing liquefied chloride of methyl, dissolved in ether, by means of a hair pencil or a medicine-dropper, as a local anæsthetic, with the best results. By i s aid he has been able to practice section of urethral strictures, open abscesses, incise the skin, and even draw teeth, without the experience of the least pain by the patient. In the latter case the only difficulty is experienced in the extraction of the last molars of the upper and lower jaws. Sometimes he has found sloughing of the mucous membrane to follow as the result of the application of the chloride of methyl; but this is rarely the case, and when it does occur is but superficial. In acute periostitis the application of chloride of methyl is often painful by implicating the neighboring teeth, and the anæsthesia is obtained with difficulty; but nevertheless the pain of the extraction of the teeth is greatly reduced. He has also employed it in opening alveolar abscesses and in various operations within the mouth. Finally, he claims that the hemorrhage, which is often troublesome after the extraction of teeth or operations upon the mouth, is really controlled by the application of liquefied chloride of methyl.—*Therapeutic Gazette.*

